# Chloroplast Genome Characterization of *Benthamiella Patagonica* (Benthamielleae, Solanaceae)

**DOI:** 10.1002/ece3.72613

**Published:** 2025-12-09

**Authors:** Abdullah, Liya Guo, Abdur Rab, Abdul Sammad, Rushan Yan, Parviz Heidari, Xiaoxuan Tian

**Affiliations:** ^1^ State Key Laboratory of Chinese Medicine Modernization Tianjin University of Traditional Chinese Medicine Tianjin China; ^2^ Haihe Laboratory of Modern Chinese Medicine Tianjin China; ^3^ Departement of Zoology Hazara University Mansehra Pakistan; ^4^ Faculty of Agriculture Shahrood University of Technology Shahrood Iran

**Keywords:** amino acid, Benthamielleae, codon usage, Nicotianoideae, Petunioideae, repeats

## Abstract

The Solanaceae is a globally distributed and economically important flowering plant family comprising approximately 2700–3000 species. Although extensive genomic studies have focused on major genera such as *Solanum* L. and *Capsicum* L., early‐diverging lineages, including Benthamielleae Hunz., remain poorly characterized. Here, we report the first complete chloroplast (cp) genome of *Benthamiella patagonica* Speg., representing Benthamielleae. The cp genome (155,570 bp) exhibits the typical quadripartite structure, comprising a large single‐copy region (LSC, 86,085 bp), a small single‐copy region (SSC, 18,445 bp), and a pair of inverted repeats (IRs, 25,520 bp each). A total of 112 unique functional genes were annotated, including 78 protein‐coding genes, 30 tRNA genes, and 4 rRNA genes, of which 18 contained introns. The *infA* and *ycf15* genes were pseudogenized, while truncated copies of *ycf1* and *rps19* were detected at the IR junctions alongside their functional counterparts. When pseudogenes and duplicated genes in IRs were included, the total gene count reached 133. Comparative analyses with representative species from three Solanaceae subfamilies revealed high similarity in genome size, gene content, and GC composition. Codon usage analysis showed an A/T bias, and simple sequence repeat analysis indicated a predominance of A/T‐rich mononucleotide repeats. Phylogenetic reconstruction based on 34 Solanaceae species and one outgroup placed *B. patagonica* as a basal lineage. The subfamily Solanoideae represented the most recently diverged clade, forming a sister group with Nicotianoideae, both rooted by Petunioideae and subsequently by *B. patagonica* (Benthamielleae). This newly sequenced cp genome fills a key gap in Solanaceae cp genomic resources. It can provide a valuable foundation for future studies on the taxonomy and evolutionary history of this lineage.

## Graphical abstract

This study presents the first complete chloroplast genome (155,570 bp) of *Benthamiella patagonica*, representing the early‐diverging tribe Benthamielleae within Solanaceae. Comparative genomic analyses revealed high structural similarity with other Solanaceae species, while phylogenetic reconstruction confirmed *B. patagonica* as a basal lineage, positioned before the divergence of the three major subfamilies. This newly sequenced genome fills a gap in Solanaceae chloroplast genomic resources and provides a valuable foundation for understanding the evolutionary history of this economically important plant family.

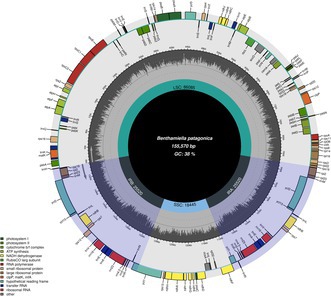

## 1 Introduction

The Solanaceae, a globally distributed and economically important family of angiosperms, comprises approximately 2700–3000 species across 102 genera and has traditionally been classified into eight subfamilies: Goetzeoideae, Cestroideae, Nicotianoideae, Solanoideae, Schizanthoideae, Schwenckioideae, Duckeodendroideae, and Petunioideae (Huang et al. 2023; WFO 2025). However, a recent PhyloCode‐based framework has proposed a rank‐free classification system for the family. This system retains traditional taxon names when associated with well‐supported clades while defining clades solely on the basis of shared ancestry and phylogenetic evidence, independent of traditional Linnaean ranks (Deanna et al. 2025). This framework is supported by a comprehensive phylogenetic analysis of 1474 species (representing 97 of the 102 genera) using 10 plastid and nuclear markers derived from the Angiosperm353 probe set. These analyses established 38 rank‐free clade names across Solanaceae to promote nomenclatural stability and minimize repeated taxonomic changes as new phylogenomic data emerge.

Within Solanaceae, the phylogenetic placement of the tribe Benthamielleae has long been a subject of taxonomic debate. Earlier treatments considered it as *incertae sedis* (Barboza et al. 2016; J. Huang et al. 2023). In the PhyloCode framework, Benthamielleae Hunziker 2000 [R.G. Olmstead & R. Deanna] is recognised as a rank‐free clade sister to Cestroideae and comprises 15 species distributed across three Patagonian genera—*Benthamiella* Speg., *Combera* Sandwith, and *Pantacantha* Speg. The group is distinguished by diagnostic apomorphies such as pollen with large, irregularly shaped exine ornamentations and two opposed leaf‐like bracteoles subtending the flowers, except in *Pantacantha* (Barboza et al. 2016; Stafford and Knapp 2006). Among these, *Benthamiella*, endemic to southern Chile and Argentina, includes 12 accepted species (POWO 2025). *Benthamiella patagonica* Speg. is native to Argentine Patagonia (Chubut and Santa Cruz provinces) and the Magallanes region of Chile. This small subshrub inhabits temperate biomes and is well adapted to arid and semi‐arid environments (POWO 2025). Resolving the phylogeny of Benthamielleae is crucial for understanding early divergences and diversification patterns within Solanaceae.

The chloroplast (cp) genome typically exhibits a conserved quadripartite structure, comprising a large single‐copy (LSC) region, a small single‐copy (SSC) region, and two inverted repeats (IRa and IRb) (Abdullah, Fatima, et al. 2025; Daniell et al. 2016; Zeng et al. 2025). Owing to its moderate level of polymorphism and predominantly maternal inheritance in angiosperms, the cp genome serves as a valuable resource for phylogenetic inference, population genetics, DNA barcoding, and genetic conservation (Daniell et al. 2016; Zhang et al. 2023). Nevertheless, complete cp genome sequences are currently unavailable for any genus within Benthamielleae.

To address this knowledge gap, the first complete cp genome assembly of *B. patagonica* is presented here. This genome provides a cp reference for the genus *Benthamiella* and the tribe Benthamielleae. The newly generated genomic resource is anticipated to facilitate future research on the evolution, taxonomy, and conservation of this underexplored lineage.

## 2 Materials and Methods

### 2.1 Sample Collection, DNA Extraction, and Sequencing

Leaf material of *B. patagonica* was obtained from herbarium specimen C.320003 housed at the Finnish Museum of Natural History, University of Helsinki, Finland. Information regarding the specimen and its representative photographs can be accessed online at https://laji.fi/en/view?uri=http:%2F%2Fid.luomus.fi%2FC.320003.

Genomic DNA was extracted from 5 mg of dried leaf tissue using the Plant Genomic DNA Kit (TIANGEN BIOTECH, Beijing, China) with several modifications: (i) 2 mL of extraction buffer (instead of 0.7 mL) was used for grinding the leaf tissue, which was divided equally into two 1.5‐mL tubes and processed as separate samples following the manufacturer's protocol; (ii) the incubation period at 65°C was extended to 90 min instead of 15 min; (iii) the supernatants from both tubes were combined and loaded onto a single DNA binding column (CD3); and (iv) DNA was eluted in 65 μL of elution buffer after extended incubation of 40 min at room temperature instead of 5 min.

DNA quality and concentration were assessed using the Agilent 5400 System (Agilent Technologies), which confirmed high‐molecular‐weight DNA at a concentration of 0.48 ng/μL (total yield: 31 ng). Paired‐end sequencing (2 × 150 bp) was subsequently performed by Novogene (Tianjin, China) on the Illumina NovaSeq 6000 platform with an average insert size of 350 bp. The quality of the raw reads was evaluated using fastp (Chen et al. 2018) following the same parameters as described previously (Y. Huang et al. 2025).

### 2.2 Chloroplast Genome Assembly, Annotation, and Feature Analysis

The cp genome was de novo assembled from whole genome sequencing data using GetOrganelle v1.7.7 (Jin et al. 2020) with default parameters and annotated using GeSeq v2.03 (Tillich et al. 2017). The start and stop codons of protein‐coding genes, as well as the annotations of all other genes, were manually curated through visual inspection and comparison with the cp genomes of *Petunia exserta* Stehmann (MT644125), *Solanum lycopersicum* L. (KP117024), *Solanum dulcamara* L. (KY863443), and *Nicotiana tabacum* L. (Z00044.2). *Solanum dulcamara* served as a particularly valuable reference due to prior transcriptomic validation of its cp genome annotations (Amiryousefi et al. 2018b). Transfer RNA (tRNA) genes were further confirmed using tRNAscan‐SE v2.0.7 (Chan and Lowe 2019).

The coverage depth and assembly accuracy were assessed by mapping raw reads back to the de novo assembled cp genome using Geneious Prime v2023.2.1 (Biomatters Ltd.) with default settings (Kearse et al. 2012). The annotated cp genome was deposited in the National Center for Biotechnology Information (NCBI) database under accession number PV928260. The genome map was generated using Chloroplot v1 (Zheng et al. 2020). Structural features were further analysed in Geneious Prime v2023.2.1. The intron content was analysed using CPGview v1 (Liu et al. 2023).

### 2.3 Comparative Analysis With Related Species

For comparative analysis, three representative Solanaceae species with well‐annotated chloroplast genomes were included: *Nicotiana tabacum* (accession no. Z00044), *Petunia exserta* (accession no. MT644125), and *Solanum lycopersicum* (accession no. KP117024). These species were selected to provide a framework for comparison with the *B. patagonica* cp genome in terms of gene content, genome organization, contraction and expansion of inverted repeats, and GC content. Genome features were analyzed in Geneious Prime, while gene arrangements were assessed using Mauve progressive alignment (Darling et al. 2004). The IR contraction and expansion were analyzed using CPJSdraw v.1 (Li et al. 2023).

### 2.4 Analysis of Codon Usage, Amino Acid Frequency, and Repeats

Relative synonymous codon usage (RSCU) and amino acid frequencies were calculated using custom Python scripts (Scripts 1 and 2). Simple sequence repeats (SSRs) were identified using MISA‐web (Beier et al. 2017) with the following minimum thresholds: mononucleotides (≥ 10), dinucleotides (≥ 5), trinucleotides (≥ 4), and tetra‐, penta‐, and hexanucleotides (≥ 3). Dispersed repeats—including forward, reverse, palindromic, and complementary types—were detected using REPuter (Kurtz et al. 2001) with a minimum repeat size of 30 bp, a maximum of 500 computed repeats, and a Hamming distance of 3.

### 2.5 Phylogenetic Analysis of Chloroplast Genomes

A maximum likelihood (ML) phylogeny was reconstructed using whole cp genome sequences of *B. patagonica* and 33 other Solanaceae species retrieved from NCBI. *Ipomoea alba* (accession ON209203.1) from the family Convolvulaceae was used as an outgroup. To avoid overrepresentation of duplicated regions, the IRa region was removed from each genome. Sequences were aligned using MAFFT (Katoh and Standley 2013) and analyzed in IQ‐TREE v3 (Minh et al. 2020). The best‐fit substitution model (TVM + F + R5) was identified using ModelFinder (Kalyaanamoorthy et al. 2017). Node support was assessed with 1000 ultrafast bootstrap replicates (Hoang et al. 2018) and SH‐aLRT tests. The resulting phylogenetic tree was visualized and annotated using the Interactive Tree of Life (Letunic and Bork 2019).

## 3 Results and Discussion

### 3.1 Sequencing of *B. patagonica*

Sequencing generated 23.75 million raw reads, corresponding to 3.56 gigabases (Gb). After quality filtering, 23.53 million clean reads (3.53 Gb) were retained, representing 99.1% of the data. The reads exhibited low error rates (0.01%), high‐quality scores (Q20 = 99.36%, Q30 = 97.36%), and a GC content of 43.37%. Despite using DNA extracted from herbarium specimens, modifications to the extraction protocol—such as utilizing smaller amounts of leaf tissue and employing a high‐efficiency isolation approach—minimized DNA degradation and yielded high‐quality data. Approximately 0.56 million reads were derived from the cp genome, providing an average coverage depth of 538×, which ensured a high‐quality and complete cp genome assembly.

### 3.2 Chloroplast Genome Structure and Gene Content of *B. patagonica* and Comparison With Related Species

**FIGURE 1:**
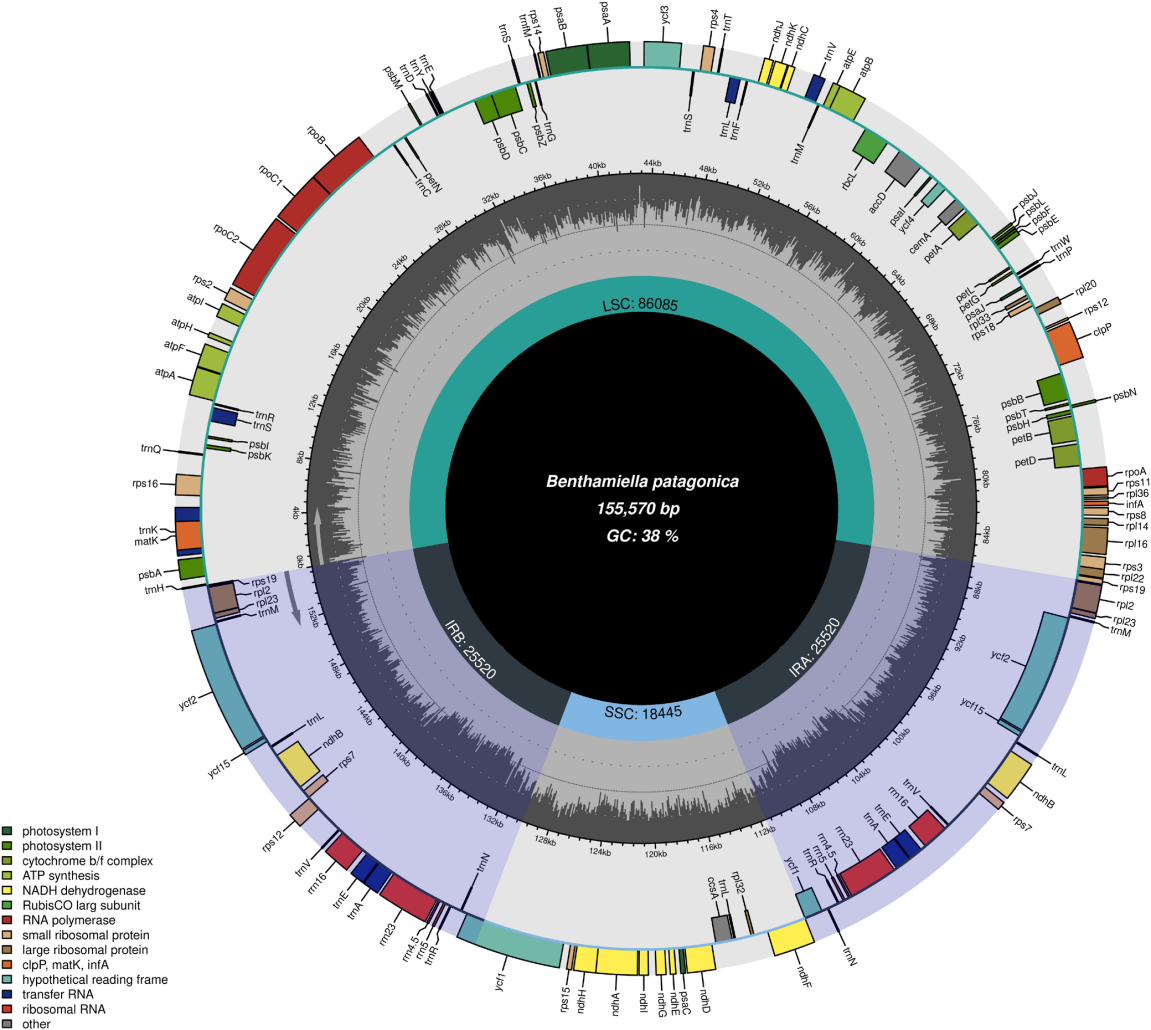
Circular representation of the chloroplast genome of *B. patagonica* . The map illustrates the major structural regions of the genome, including the large single‐copy (LSC) region, small single‐copy (SSC) region, and two inverted repeat regions (IRa and IRb). Genes are annotated around the circle and colour‐coded according to their functional categories, as indicated in the legend (e.g., photosystem, ribosomal proteins, RNA polymerase, etc.). Genes located on the outer circle are transcribed in the anticlockwise direction, while those on the inner circle are transcribed clockwise. The inner grey histogram represents the GC content across the genome, with an average GC content of approximately 38%. The total length of the chloroplast genome is 155,570 base pairs (bp).

**FIGURE 2:**
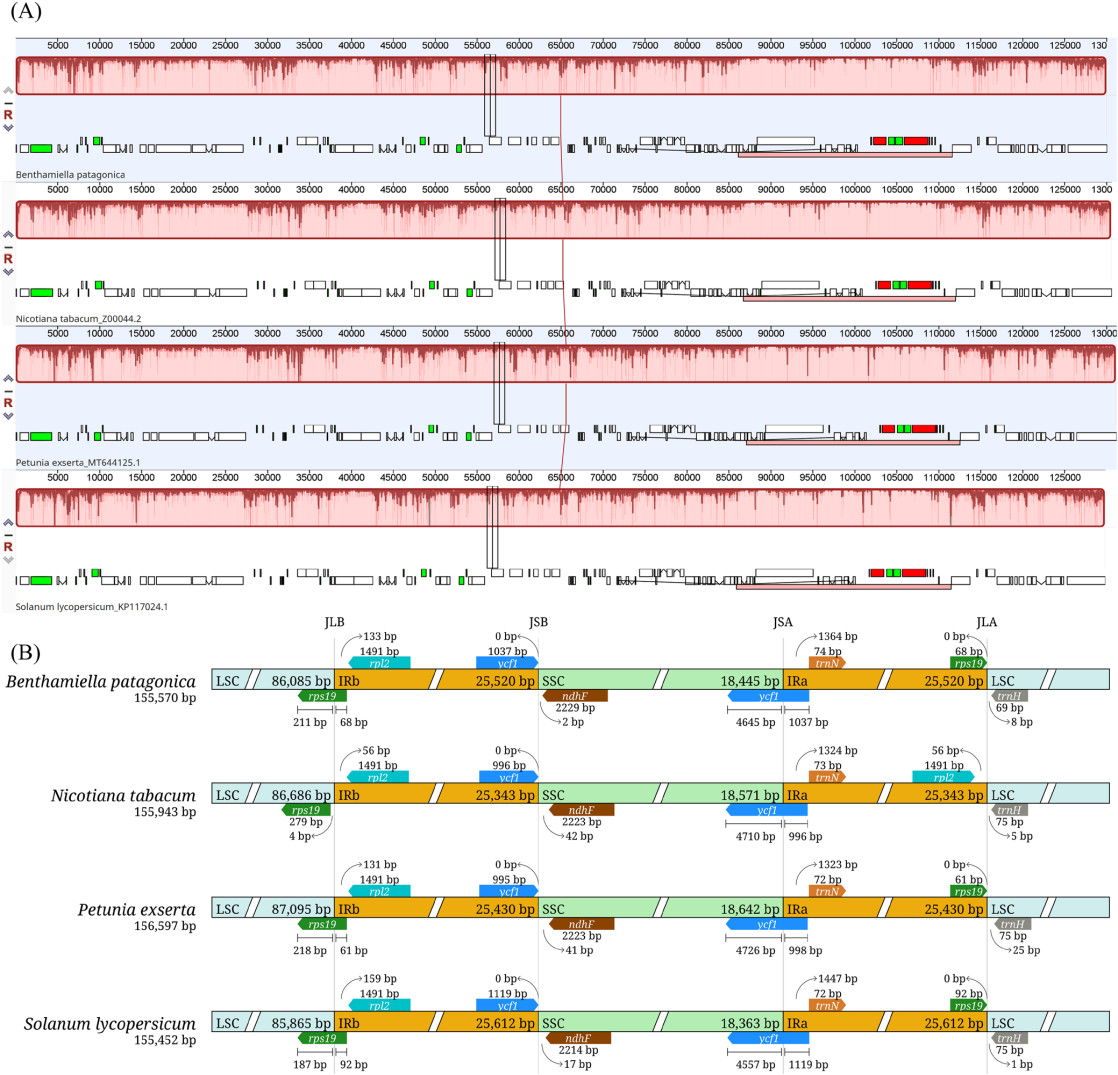
Comparison of gene arrangements among selected Solanaceae species. (A) Mauve progressive alignment indicates high conservation of gene arrangement without any inversion that affects gene arrangement. (B) The inverted repeat boundaries show high conservation among species at four junctions, including LSC/IRb (JLB), IRb/SSC (JSB), SSC/IRa (JSA), and IRa/LSC (JLA).

The *B. patagonica* cp genome, like other Solanaceae species, exhibited the typical quadripartite structure, consisting of LSC, SSC, and two IRs (Figure 1). It contained 112 unique functional genes, comprising 78 protein‐coding genes, 30 tRNA genes, and 4 rRNA genes (Table 1). This count excluded *infA* and *ycf15*, which are pseudogenes in all four compared species ( *B. patagonica* , *N. tabacum* , *P. exserta* , and *S. lycopersicum* ). Partial copies of *rps19* and *ycf1* at the IR junctions were also excluded from the functional gene count; one functional copy of each gene was present in the genome. The IRs contained duplicated genes essential for ribosomal function and genome stability, including the four rRNA genes (*rrn16*, *rrn23*, *rrn4.5*, *rrn5*), seven tRNA genes (*trnI‐GAU*, *trnA‐UGC*, *trnR‐ACG*, *trnN‐GUU*, *trnV‐GAC*, *trnL‐CAA*, *trnI‐CAU*), six protein‐coding genes (*rpl2*, *rpl23*, *rps7*, *ndhB*, *ycf2*), and the *ycf15* pseudogene. The 3′ exons of the trans‐spliced *rps12* gene were present in the IRs, but the gene was not considered duplicated. When duplicated and non‐functional genes were included, the total gene count rose to 133 (Table 1). All four species showed similar gene content and arrangement, except that the *rps19* pseudogene was missing in *Nicotiana tabacum* (Figure 2A). Gene localisation followed the conserved Solanaceae pattern: the LSC harboured the majority of protein‐coding genes (e.g., *rbcL*, *atpB*, *atpE*, *rpoB*, *rpoC1*, *rpoC2*) and several tRNAs, whereas the SSC was predominantly composed of *ndh* genes (*ndhD*, *ndhE*, *ndhF*, *ndhG*, *ndhI*). This organisation and genome size were consistent across all four species and with cp genomes reported for other Solanaceae and dicotyledonous plants (Abdullah, Fatima, et al. 2025; Amiryousefi et al. 2018a; He et al. 2024; Mehmood, Abdullah Shahzadi, et al. 2020; Mehmood, Abdullah, et al. 2020). Pseudogenization of *infA* is a common evolutionary event in angiosperms, including Solanaceae, often associated with its functional transfer to the nucleus (Abdullah Mehmood et al. 2020; Mehmood, Abdullah, et al. 2020; Millen et al. 2001; Tian et al. 2018).

**TABLE 1:** Genetic content of *B. patagonica* .

Category for genes	Group of genes	Name of genes	Amount
Self‐replication	Large subunit of ribosome	*rpl14*, *rpl16* ^a^, *rpl2* ^a^, ^b^, *rpl20*, *rpl22*, *rpl23* ^b^, *rpl32*, *rpl33*, *rpl36*	11
	Small subunit of ribosome	*rps11*, *rps12* ^a^, *rps14*, *rps15*, *rps16* ^a^, *rps18*, *rps19*, *rps2*, *rps3*, *rps4*, *rps7* ^b^, *rps8*	13
	DNA dependent RNA polymerase	*rpoA*, *rpoB*, *rpoC1* ^a^, *rpoC2*	4
	rRNA genes	*rrn16* ^b^, *rrn23* ^b^, *rrn4.5* ^b^, *rrn5* ^b^	8
	tRNA genes	*trnA‐UGC* ^a^, ^c^, *trnC‐GCA*, *trnD‐GUC*, *trnE‐UUC* ^a^, ^b^, *trnE‐UUG* ^a^, *trnF‐GAA*, *trnG‐GCC*, *trnH‐GUG*, *trnK‐UUU* ^a^, *trnL‐CAA* ^b^, *trnL‐UAA* ^a^, *trnL‐UAG*, *trnI‐CAU* ^b^, *trnN‐GUU* ^b^, *trnP‐UGG*, *trnQ‐UUG*, *trnR‐ACG* ^b^, *trnR‐UCU*, *trnS‐CGA* ^a^, *trnS‐GCU*, *trnS‐GGA*, *trnS‐UGA*, *trnT‐GGU*, *trnT‐UGU*, *trnV‐UAC* ^a^, *trnV‐GAC* ^b^, *trnW‐CCA*, *trnY‐GUA*, *trnfM‐CAU*	37
Photosynthesis	Photosystem I	*psaA*, *psaB*, *psaC*, *psaI*, *psaJ*	5
	Photosystem II	*psbA*, *psbB*, *psbC*, *psbD*, *psbE*, *psbF*, *psbH*, *psbI*, *psbJ*, *psbK*, *psbL*, *psbM*, *psbN (psfI)*, *psbT*, *psbZ*	15
	NADPH dehydrogenase	*ndhA* ^a^, *ndhB* ^a^, ^b^, *ndhC*, *ndhD*, *ndhE*, *ndhF*, *ndhG*, *ndhH*, *ndhI*, *ndhJ*, *ndhK*	12
	Photosystem I assembly proteins	*ycf3* ^a^ *(paf1)*, *ycf4 (pafII)*	2
	Cytochrome b/f complex	*petA*, *petB* ^a^, *petD* ^a^, *petG*, *petL*, *petN*	6
	Subunits of ATP synthase	*atpA*, *atpB*, *atpE*, *atpF* ^a^, *atpH*, *atpI*	6
	Large subunit of Rubisco	*rbcL*	1
Other genes	Protease	*clpP* ^a^	1
	Maturase	*matK*	1
	Envelop membrane protein	*cemA*	1
	Subunit of Acetyl‐CoA‐carboxylase	*accD*	1
	C‐type cytochrome synthesis gene	*ccsA*	1
	Translation initiation factor	*infA* ^c^	1
	Conserved open reading frames	*ycf1*, *ycf15* ^b^, ^c^, *ycf2* ^b^	5
	Pseudogene fragments	*ycf1* ^c^, *rps19* ^c^	2
Total number of genes			133

^a^ Intron containing genes.

^b^ Duplicated genes in inverted repeats.

^c^ Pseudo genes fragments present at inverted repeat junctions.

### 3.3 Comparison of Genome Size, GC Content, and IR Boundaries

The 155,570 bp cp genome of *B. patagonica* exhibited the typical quadripartite structure, consisting of LSC (86,085 bp), SSC (18,445 bp), and two IRs (25,520 bp each) (Figure 1 and Table 2). The overall genome length was comparable to those of *Nicotiana tabacum* (155,943 bp), *Petunia exserta* (156,597 bp), and *Solanum lycopersicum* (155,452 bp). Similarly, the sizes of LSC (86,085–87,095 bp), SSC (18,363–18,642 bp), and IR (25,343–25,612 bp) regions showed minimal variation among these species, indicating strong structural conservation across Solanaceae (Table 2).

**TABLE 2:** Genome feature comparisons of *B. patagonica* with other related species.

Species		*B. patagonica*	*N. tabacum*	*P. exserta*	*S. lycopersicum*
Genome length (bp)		155,570	155,943	156,597	155,452
LSC length (bp)		86,085	86,686	87,095	85,865
SSC length (bp)		18,445	18,571	18,642	18,363
IR length (bp)		25,520	25,343	25,430	25,612
GC content (%)	Total	37.79	37.85	37.81	37.86
	Large‐single copy	35.86	35.95	35.93	35.99
	Small‐single copy	31.78	32.07	32.07	32.03
	Inverted repeats	43.21	43.22	43.14	43.09
	Transfer RNA	52.64	52.85	53	52.97
	Ribosomal RNA	55.28	55.37	55.34	55.33
	Protein‐coding genes	38.12	38.19	38.22	38.26
Accession Number		PV928260	Z00044	MT644125	KP117024

The total GC content of *B. patagonica* (37.79%) was similar to that of *N. tabacum* (37.85%), *P. exserta* (37.81%), and *S. lycopersicum* (37.86%). In all four species, the IRs exhibited the highest GC proportion (43.09%–43.22%) due to the abundance of rRNA genes, whereas the SSC showed the lowest GC content (31.78%–32.07%). tRNA (52.64%–53%) and rRNA (55.28%–55.37%) genes displayed higher GC content than coding sequences (38.12%–38.26%), reflecting typical compositional patterns of Solanaceae cp genomes (Table 2).

Examination of IR boundaries revealed a highly conserved organisation across all four species. The LSC/IRb junction (JLB) consistently included *rpl2* within the IR, while *rps19* spanned the junction in all species except *Nicotina tabacum* , where it was entirely located in the LSC. The IRb/SSC junction (JSB) contained a functional *ycf1*, while the SSC/IRa junction (JSA) harboured its truncated pseudogene (*ycf1Ψ*, 996–1119 bp). At the IRa/LSC junction (JLA), *trnH‐GUG* was located in the LSC, and a short *rps19* pseudogene (61–92 bp) was present on the IRa side in all species except *N. tabacum* . The *ndhF* gene was entirely located in the SSC in all taxa (Figure 2B).

This conserved organisation of IR boundaries, along with minimal variation in genome size and GC content, highlights the evolutionary stability of Solanaceae cp genomes. Despite reports of IR contraction and expansion at the subfamily level in other dicots (Abdullah, Haram, et al. 2025) and gene rearrangements at the family level (Abdullah, Li, et al. 2025), all four species analysed here exhibited a highly conserved genome architecture. This observation reinforces the notion that the Solanaceae cp genome is structurally stable.

These findings align with previous observations in Solanaceae and other plant lineages (Amiryousefi et al. 2018a; He et al. 2024; Mehmood, Abdullah, et al. 2020), suggesting that slow cp genome evolution, gene organisation in operon, active DNA repair systems, uniparental inheritance, and rare plastid fusion collectively contribute to the maintenance of structural and compositional conservation (Wicke et al. 2016).

### 3.4 Analysis of Intron‐Containing Genes

**FIGURE 3:**
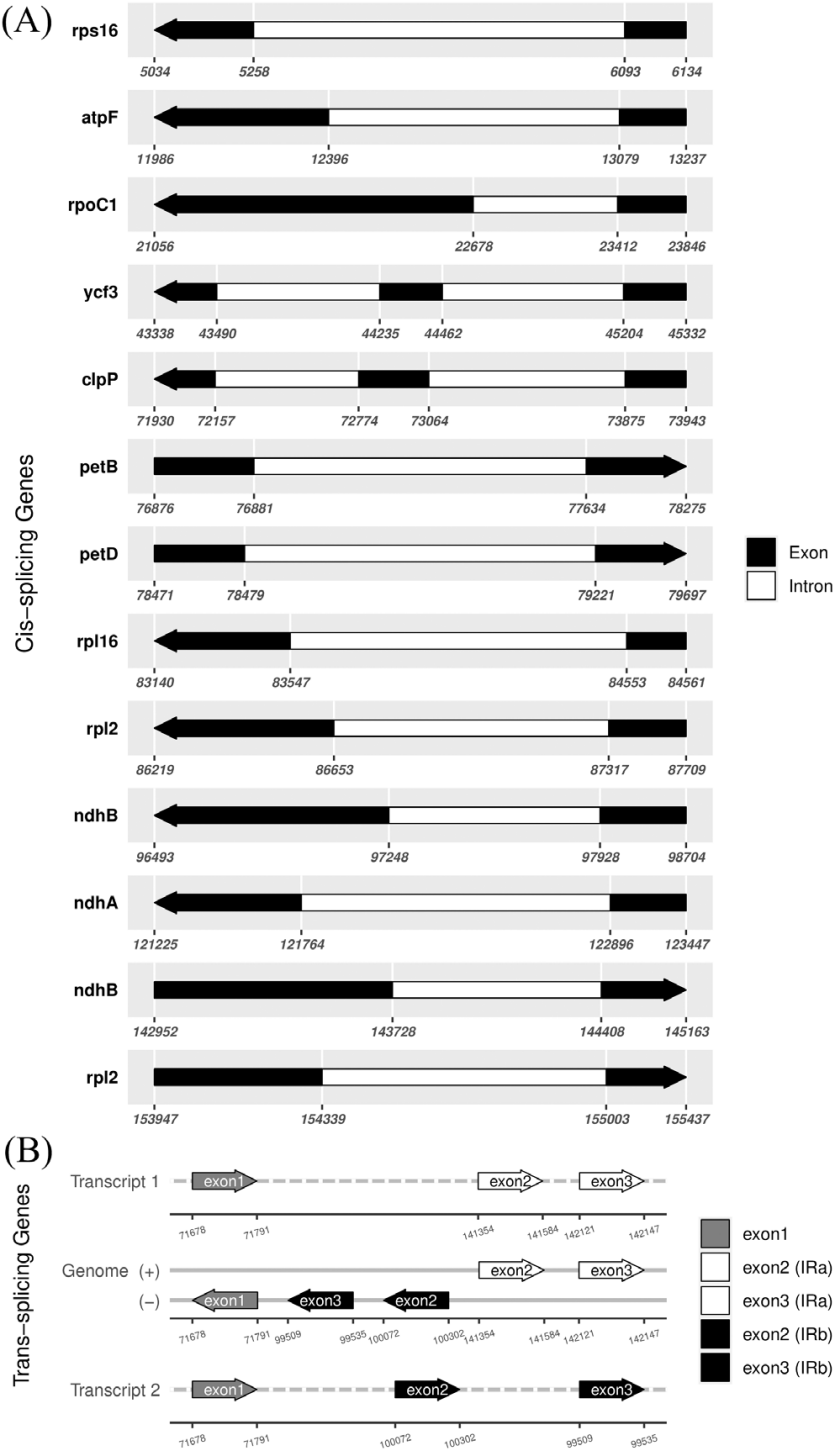
Structures of intron‐containing genes in the *B. patagonica* chloroplast genome. (A) Schematic diagrams of cis‐spliced genes, showing the arrangement of exons (black boxes) and introns (white boxes), with gene names labelled at the left side of each diagram. Arrows of each diagram indicate the direction of transcription. (B) Schematic diagram of the trans‐spliced *rps12* gene, which has its 5′ exon located in the LSC region and two duplicated 3′ exons located in the IRa and IRb regions. The mature RNA transcripts produced after splicing (Transcript 1 and Transcript 2) are also illustrated.

The *B. patagonica* cp genome encodes 18 intron‐containing genes: 17 cis‐splicing genes and one trans‐splicing gene, *rps12* (Figure 3A,B). Among the cis‐spliced genes, 15 contain a single intron, while *clpP* and *ycf3* each contain two introns (Figure 3A). These genes represent multiple functional categories, including ribosomal proteins (*rpl2*, *rpl16*, *rps16*), NADH dehydrogenase subunits (*ndhA*, *ndhB*), photosystem components (*petB*, *petD*), ATP synthase (*atpF*), RNA polymerase (*rpoC1*), and protease (*clpP*).

All cis‐spliced genes adhere to the canonical exon–intron structure. The multi‐intronic genes *clpP* and *ycf3* consist of three exons, reflecting more complex splicing requirements.

The trans‐splicing gene *rps12* exhibited a distinctive architecture: its 5′ exon (exon 1) resided in the LSC region, whereas the 3′ exons (exons 2 and 3) were duplicated within the IRs (Figure 3B). This spatial separation necessitates post‐transcriptional assembly of the mature mRNA, a fundamental and conserved mechanism in angiosperm cp genomes. Figure 3B illustrates the locations of *rps12* exons and their assembly into transcript isoforms 1 and 2, demonstrating this mechanistic complexity.

Collectively, these findings confirm that the *B. patagonica* cp genome possesses the typical complement and organization of intron‐containing genes characteristic of Solanaceae and angiosperms in general (Liu et al. 2025; Mehmood, Abdullah Shahzadi, et al. 2020; Mehmood, Abdullah, et al. 2020; Nhat Nam et al. 2025; Vo‐Tan et al. 2025).

### 3.5 Relative Synonymous Codon Usage (RSCU) Patterns and Amino Acid Frequency

**FIGURE 4:**
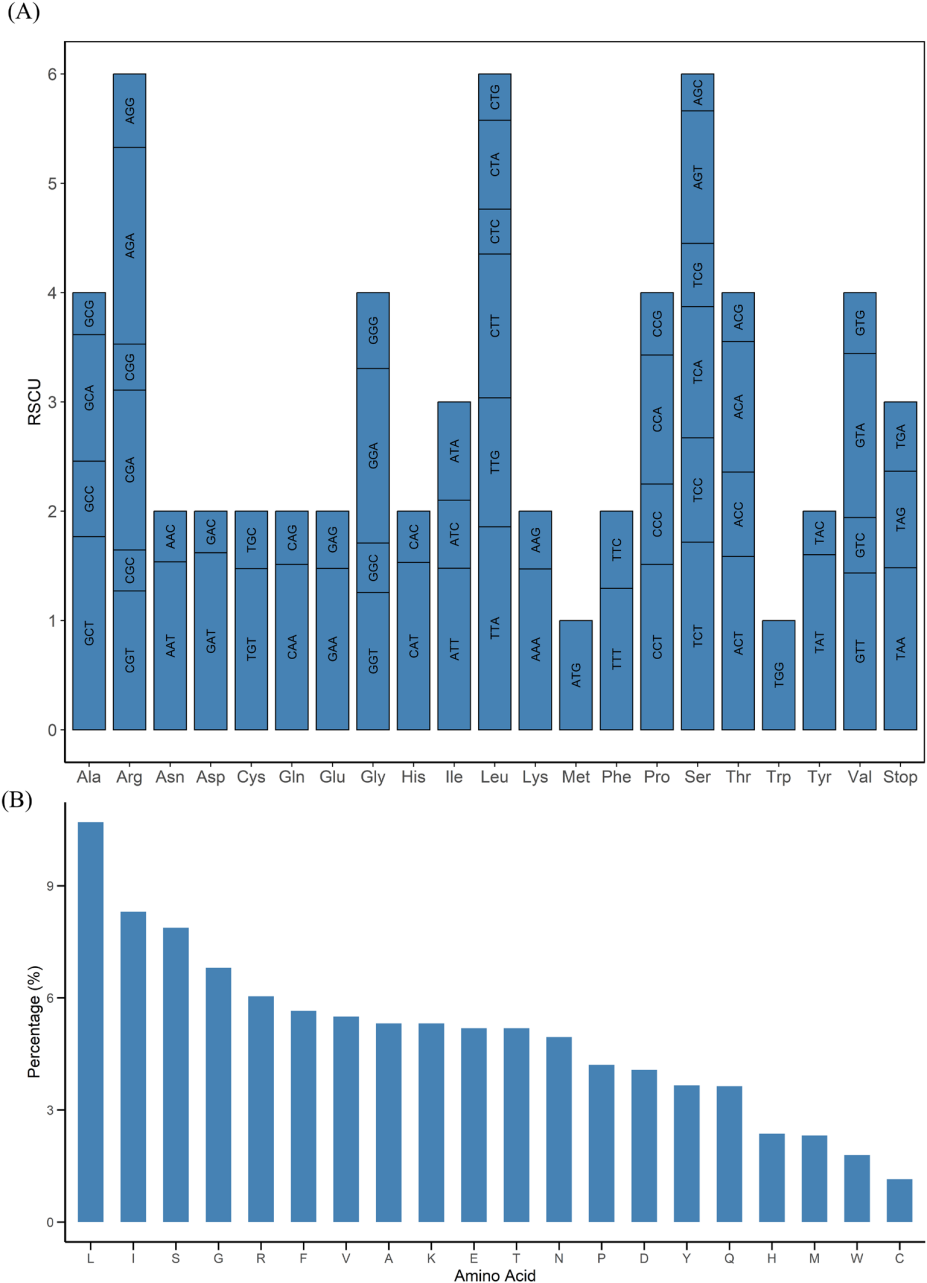
Relative synonymous codon usage and amino acid frequency analysis of protein‐coding genes of the *B. patagonica* chloroplast genome. (A) Codon usage analysis. The x‐axis represents amino acids (e.g., Ala, Arg, Asn, Asp, etc.), and each stacked bar corresponds to the synonymous codons for that amino acid, with the codon identities labeled within the bars. The y‐axis shows the relative synonymous codon usage (RSCU) values, which reflect how frequently a codon is used compared to uniform usage. A dashed line at RSCU = 1 indicates no bias; codons with RSCU > 1 are used more frequently and are considered preferred codons. (B) Amino acid composition. *B. patagonica* . The x‐axis displays amino acids using their one‐letter abbreviations (e.g., L for leucine, I for isoleucine, S for serine, etc.). The y‐axis indicates the relative frequency of each amino acid as a percentage of the total amino acids encoded by the genome. Leucine (L), isoleucine (I), and serine (S) are the most abundant amino acids, while cysteine (C) is the least abundant.

Analysis of relative synonymous codon usage (RSCU) in protein‐coding genes revealed a pronounced bias towards codons ending in A or T (Figure 4A). For example, among codons encoding alanine, GCT showed a notable preference (RSCU = 1.77) compared to GCG (RSCU = 0.39). An RSCU value greater than 1 indicates preferential usage, while values below 1 indicate underrepresentation.

Amino acid frequencies in the *B. patagonica* cp genome varied considerably (Figure 4B). Leucine dominated at 10.70% of residues, followed by isoleucine (8.30%), serine (7.88%), and glycine (6.81%). Arginine, phenylalanine, valine, and alanine each exceeded 5% abundance. In contrast, cysteine and tryptophan were the rarest residues (1.14% and 1.79%, respectively).

This pervasive AT‐skewed codon usage and amino acid frequency align with patterns observed across land plant cp genomes (Abdullah, Fatima, et al. 2025; Mehmood, Abdullah Ubaid, et al. 2020; Mehmood, Abdullah, et al. 2020; Yang et al. 2019) and primarily reflect mutational pressures and translational selection within the cp genome.

### 3.6 Characterization of Simple Sequence Repeats and Oligonucleotide Repeats

**FIGURE 5:**
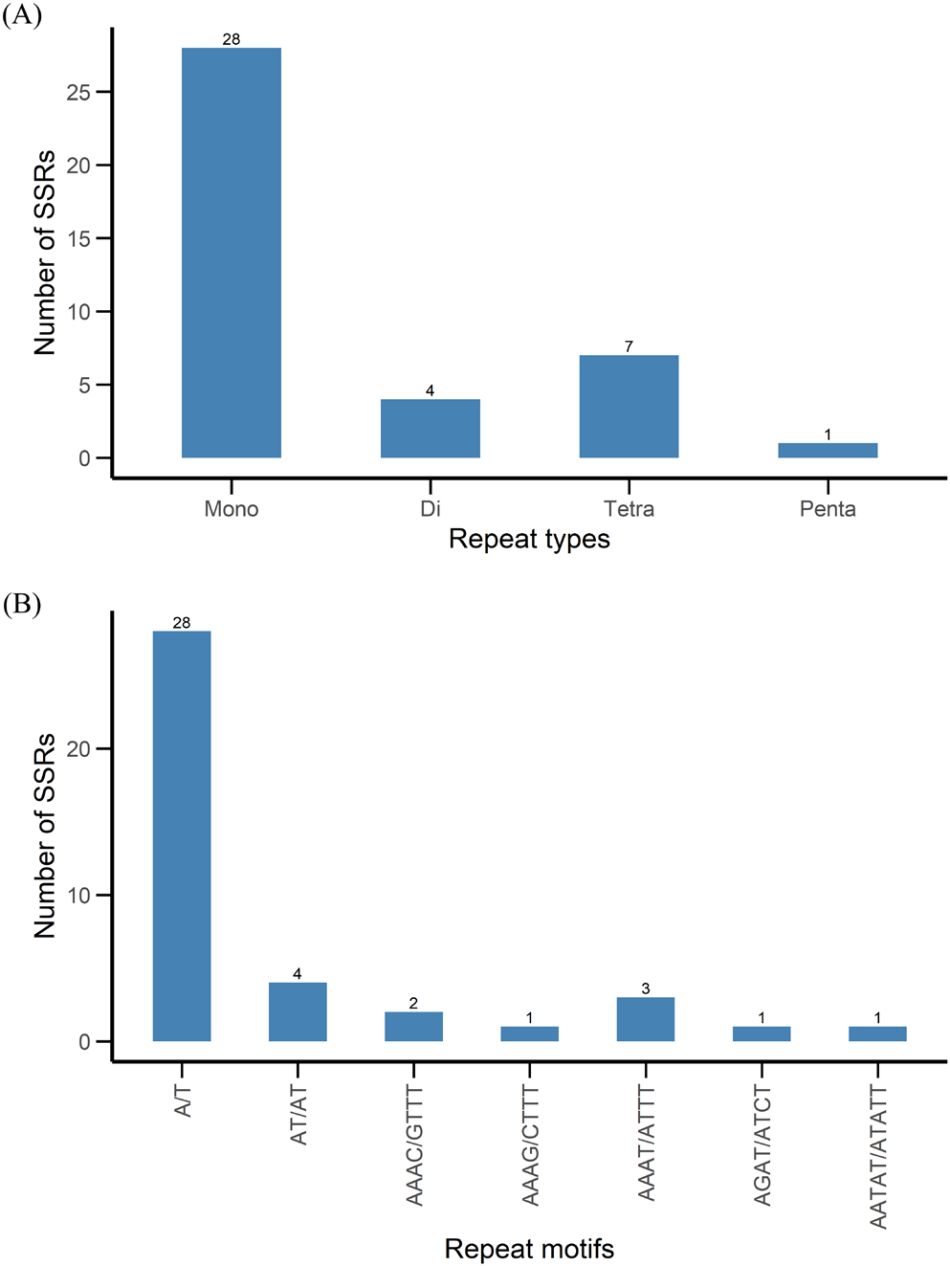
Distribution of simple sequence repeats (SSRs) in the chloroplast genome of *B. patagonica* . (A) Frequency of SSRs categorized by repeat type. The x‐axis lists the types of SSRs detected: Mononucleotide, dinucleotide, tetranucleotide, and pentanucleotide repeats. The y‐axis represents the number of SSRs identified in each category. The counts of SSRs per category are also labeled above the bars. (B) Frequency of SSRs based on specific repeat motifs. The x‐axis shows the identified repeat motifs (e.g., A/T, AT/AT, AAAC/TTTT, etc.), and the y‐axis indicates the number of SSRs corresponding to each motif. The most abundant motif observed is the mononucleotide A/T repeat. Numbers above each bar indicate the count of SSRs for each motif.

A comprehensive analysis identified 40 SSRs in the *B. patagonica* cp genome, representing four types of repeats (Figure 5A). Mononucleotide repeats were the most abundant (70%, *n* = 28), followed by tetranucleotide (17.5%, *n* = 7), dinucleotide (10%, *n* = 4), and pentanucleotide (2.5%, *n* = 1) repeats. Notably, no tri‐ or hexanucleotide repeats were detected. The high prevalence of A/T‐rich mononucleotide SSRs reflects the cp genome's inherent AT bias, a common feature of angiosperm cp genomes (Abdullah, Fatima, et al. 2025; Mehmood, Abdullah, et al. 2020; Zeng et al. 2025). Motif analysis confirmed that all 28 mononucleotide repeats were predominantly composed of A/T homopolymers (Figure 5B). Dinucleotide repeats consisted exclusively of the AT/TA motif (*n* = 4). Tetranucleotide motifs included AAAC/GTTT, AAAG/CTTT, AAAT/ATTT, and AGAT/ATCT, while a single pentanucleotide repeat (AATAT/ATATT) was detected. These patterns align closely with trends observed in other Solanaceae cp genomes (He et al. 2024; Mehmood, Abdullah Ubaid, et al. 2020; Wang et al. 2022). These SSRs may serve as useful molecular markers for species delimitation, population genetics, and phylogeographic research.

Using REPuter, 17 oligonucleotide repeats ranging from 30 to 50 bp in length were identified in the cp genome of *B. patagonica* (Table 3). Only palindromic and forward repeats were present, while complementary and reverse repeats were absent. Most repeats were concentrated in the LSC region, with fewer occurrences in the IRs and SSC (Table 3). Functionally, they were primarily located within intergenic spacers (e.g., *trnE‐trnT*, *psaB/psaA*, *ndhF‐rpl32*) and intronic regions. Repeats resulting from tRNA gene duplication (e.g., *trnG* and *trnS*) were excluded to avoid overestimation. These repetitive elements are known to act as hotspots of genomic instability, frequently contributing to structural variation and increasing the rate of substitutions and insertion–deletion events, as reported in comprehensive studies of the plant families Araceae and Malvaceae (Abdullah et al. 2021; Abdullah, Mehmood, et al. 2021).

**TABLE 3:** Oligonucleotide repeats in *B. patagonica* .

Repeat size	Repeat type	Location in chloroplast	Genetic region
36	P	LSC	*trnE‐trnT*
33	P	LSC	*trnT‐psbD*
47	F	LSC	*psaB/psaA*
39	F	LSC/IR	*ycf3 intron/rps12‐trnV*
39	F	LSC/SSC	*ycf3 intron/ndhA intron*
38	P	LSC	*trnT‐trnL*
43	P	LSC	*ndhC‐trnV*
30	F	LSC	*rpl20‐rps12*
48	P	LSC	*psbT‐psbN*
50	P	LSC	*petD intron*
30	F	IR	*ycf2*
40	F	IR	*ycf2*
40	P	IR	*ycf15‐trnL*
35	P	IR	*ycf15‐trnL*
41	F	IR/SSC	*rps12‐trnV/ycf3 intron*
30	F	IR/SSC	*rps12‐trnV/ndhF‐rpl32*
37	P	SSC	*ndhF‐rpl32*

Abbreviations: F, forward repeats; IR, inverted repeats; LSC, large single copy; *P*, palindromic repeats; SSC, small single copy.

### 3.7 Phylogenetic Analysis

**FIGURE 6:**
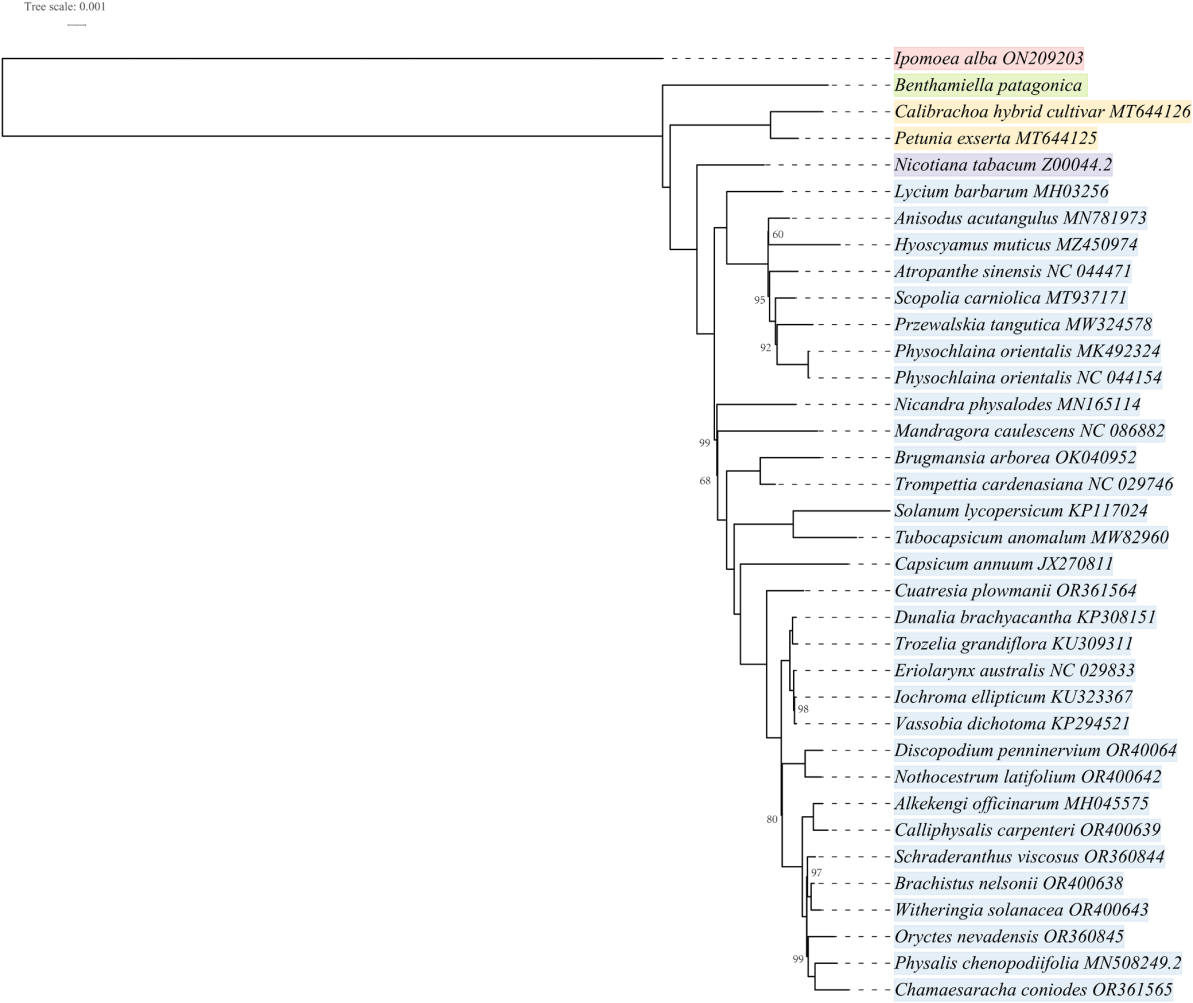
Maximum likelihood phylogenetic tree illustrating the evolutionary relationships between *B. patagonica* and 33 other Solanaceae species, based on their complete chloroplast genome sequences. *Ipomoea alba* (highlighted in orange) was the outgroup to root the tree. Taxon names are coloured according to their respective subfamilies or clades for improved visualisation: Petunioideae in yellow, Nicotianoideae in light purple, Solanoideae in blue, and *B. patagonica* , sequenced in the current study, highlighted in green to represent Benthamielleae. Bootstrap values equal to 100% for internal nodes within a subfamily member are omitted for clarity, except for the branch support of the subfamilies. The tree topology depicts the phylogenetic placement of *B. patagonica* within Solanaceae.

Our cp phylogeny robustly resolved relationships among the three major Solanaceae subfamilies and the Benthamielleae clade. Solanoideae (*Capsicum*, *Solanum*, *Physalis* L.), a species‐rich lineage, formed the most recently diverged clade and was sister to Nicotianoideae ( *N. tabacum* ). These two subfamilies were grouped with Petunioideae ( *P. exserta* , *Calibrachoa hybrid*). The newly sequenced *B. patagonica* occupied the basal position, representing the deepest divergence within Solanaceae among the understudied species. All major nodes received strong bootstrap support (100%) except for *B. patagonica* , which showed a lower bootstrap value of 76%, reflecting limited taxon sampling at basal nodes (Figure 6). Our analysis lacked representatives from four earlier‐diverging subfamilies—Schwenckioideae, Schizanthoideae, Goetzeoideae, and Duckeodendroideae—which form successive sister lineages to the core Solanaceae in broader phylogenomic analyses (Huang et al. 2023; WFO 2025). Inclusion of these taxa could alter the basal placement within the family, as previous studies suggest Goetzeoideae (Deanna et al. 2025) or Schizanthoideae occupy the most basal position (Huang et al. 2023). The relatively low bootstrap value for *B. patagonica* may reflect this taxonomic gap. Recent analyses also propose excluding *B. patagonica* from Cestroideae (Deanna et al. 2025), positioning it as basal to Petuniina (Petunioideae), with Benthamielleae forming the basal lineage. This under‐sampling limits the resolution of the family's deepest branches and obscures key early evolutionary transitions. Future studies should prioritise cp genome sequencing of these under‐represented subfamilies to improve comparative genomic and phylogenetic insights.

## 4 Conclusion

In this study, we characterized the chloroplast genome of *B. patagonica* , representing the first complete cp genome from the tribe Benthamielleae. This genomic resource provides a valuable foundation for future evolutionary, taxonomic, and phylogenetic studies within the Solanaceae. Moreover, the availability of this genome may support research on the conservation and adaptive potential of this Patagonian endemic lineage. Expanding sampling across underrepresented subfamilies may further enhance our understanding of Solanaceae evolution.

## Author Contributions

**Abdullah:** conceptualization (equal), data curation (equal), formal analysis (equal), methodology (equal), software (equal), visualization (equal), writing – original draft (equal), writing – review and editing (equal). **Liya Guo:** formal analysis (equal), writing – original draft (equal). **Abdur Rab:** data curation (equal), formal analysis (equal). **Abdul Sammad:** formal analysis (equal), investigation (equal), methodology (equal). **Rushan Yan:** data curation (equal), formal analysis (equal). **Parviz Heidari:** conceptualization (equal), investigation (equal), methodology (equal), writing – review and editing (equal). **Xiaoxuan Tian:** conceptualization (equal), investigation (equal), methodology (equal), resources (lead), writing – review and editing (equal).

## Funding

The authors have nothing to report.

## Conflicts of Interest

The authors declare no conflicts of interest.

## Data Availability

The raw sequencing data and BioSample information are available under BioProject ID PRJNA1290066. The de novo assembled cp genome is publicly available in NCBI under accession number PV928260.
